# Understanding Selective Mutism in Very Young Children

**DOI:** 10.3390/bs15070923

**Published:** 2025-07-09

**Authors:** Kimberly Renk, Kaitlyn Daleandro, Madison Verdone, Haifa Al-Bassam, Quiyara Murphy

**Affiliations:** Department of Psychology, University of Central Florida, 4000 Central Florida Blvd., Orlando, FL 32816, USA

**Keywords:** selective mutism, young children

## Abstract

Although professionals who work with children and adolescents are well aware of psychological symptom presentations once children and adolescents are in school, such symptom presentations in very young children are less understood. Diagnoses like selective mutism may promote further complications for professionals, as the symptom presentation of anxiety and failure to speak in this diagnosis may overlap with the acquisition of speech and language milestones and problems in very young children. Thus, providing professionals who work with very young children a way to adapt their thinking about selective mutism symptom presentations and interventions is of utmost importance. As a result, this clinically oriented paper will compare *DSM-5-TR* criteria to *DC:0-5* criteria, consider the occurrence of selective mutism symptoms in the context of young children’s speech and language milestones and problems, and reflect upon how intervention adaptations meant to incorporate parents into treatment and account for the capacities of very young children can be helpful in facilitating successful outcomes. It is hoped that having this constellation of clinical information in one place will help providers gain clarity regarding selective mutism symptom presentation and relevant intervention considerations for very young children.

## 1. Understanding Selective Mutism in Very Young Children

Professionals who work with children and adolescents are often well aware of psychological symptom presentations once children and adolescents are in school. Nonetheless, these same professionals may have much less understanding of such symptom presentations in very young children. There are likely many reasons for this lack of understanding. For example, although research has suggested that psychological symptoms have origins in early childhood (Rutter et al., 2006), the validity of psychological diagnoses in very young children has been questioned by some (Skovgaard et al., 2004). Further, the epidemiology of psychological diagnoses in very young children is under-researched. Studies that have been conducted described increasing incidence rates and complex comorbidities of psychological diagnoses in very young children, however (Koch et al., 2021).

Diagnoses like selective mutism may offer professionals further complications. Although many have provided reviews relevant to understanding selective mutism (e.g., Krysanski, 2003; Rozenek et al., 2020; Wong, 2010), little attention has been given to the symptom presentation of selective mutism in very young children. With selective mutism in particular, the symptom presentation of anxiety and failure to speak may overlap with young children’s acquisition of speech and language milestones and problems. This combination may make differential diagnosis potentially very difficult. Thus, providing professionals who work with very young children a way to adapt their thinking about symptom presentation and intervention is of utmost importance. As a result, this paper will compare *DSM-5-TR* criteria to the less well known *DC:0-5* criteria, consider the occurrence of selective mutism symptoms in the context of young children’s speech and language milestones and problems, and reflect upon how intervention adaptations meant to incorporate parents into treatment and account for the capacities of very young children can be helpful in facilitating successful outcomes. Each topic will be addressed in turn.

## 2. *DSM-5-TR* Criteria for Selective Mutism

Although the *International Classification of Diseases-11th Edition* (*ICD*; World Health Organization, 2018) and the *Diagnostic and Statistical Manual of Mental Disorders-Fifth Edition-Text Revision* (*DSM-5-TR*; APA, 2022) were developed in tandem for the purpose of harmonizing content, the *DSM-5-TR* is further examined here due to its common usage for psychological diagnoses. In the *DSM-5-TR*, selective mutism is specified as an anxiety disorder. Selective mutism is characterized by a child’s persistent failure to speak in social situations where there is an expectation for speaking. Based on the *DSM-5-TR* criteria, the child does not speak in select social situations despite speaking in other, often more familiar situations (APA, 2022). Thus, selective mutism is not about an individual’s lack of ability to speak but about the anxiety that they experience in specific situations. From a diagnostic perspective, the disturbance in the child’s speech must interfere with education- or occupation-related accomplishments or with social communication for at least one month. This time frame does not include the first month of school, during which time many children may be naturally reluctant to speak. In addition, selective mutism would not be diagnosed in cases where a child lacks comfort with or knowledge of the language being spoken in a particular setting (APA, 2022). Finally, the child’s failure to speak is not better explained by communication difficulties (e.g., stuttering) and is not specifically a result of a developmental or psychotic disorder, even if such issues are co-occurring. Children with selective mutism often use other ways of communicating nonverbally, such as writing or pointing (APA, 2022).

Although selective mutism was first designated as an anxiety disorder in *DSM-5* (APA, 2013), variants of this diagnosis have been under consideration for some time. The concept of selective mutism was first described by the German physician Adolf Kussmaul (1877) as aphasia voluntaria, with the assumption that children were voluntarily withholding speech in social settings. Swiss child psychiatrist Moritz Tramer (1934) began using the label ‘elective mutism,’ maintaining the idea that children were electing not to speak in certain situations. ‘Elective mutism’ was used in the *DSM* series until the *DSM-IV* (APA, 1994), when the terminology was updated to ‘selective mutism.’ With this update, the *DSM-IV* highlighted anxiety in response to certain situations or contexts as the reason for withholding speech. This suspected etiology was a break from historical definitions that assumed that the withholding of speech was solely oppositional. Thus, *selective* mutism extends the withholding of speech beyond defiance and includes the possibility of shyness, fear, or anxiety (Viana et al., 2009).

There is some evidence of comorbidity with conduct and oppositional behaviors (e.g., stubbornness, temper tantrums, negativism); children with selective mutism often exhibit a variety of anxious behaviors (e.g., excessive shyness, social anxiety, fear of public speaking; APA, 2022; Dilberto & Kearney, 2016; Vogel et al., 2024). Consistently, latent class analyses have suggested that anxiety is a large part of selective mutism presentations. For example, Dilberto and Kearney (2018) found three latent class profiles, including a moderately anxious, oppositional, and inattentive profile; a highly anxious and moderately oppositional and inattentive profile; and a mildly to moderately anxious and mildly oppositional and inattentive profile. Children in the highly anxious and moderately oppositional and inattentive profile exhibited the most impairment as well as the most emotionality, shyness, and social problems (Dilberto & Kearney, 2018).

When considering these presentations, it should be noted that children with selective mutism tend to display more impulsive and oppositional behaviors at home than in any other setting (e.g., school; Cunningham et al., 2004). Nonetheless, selective mutism is etiologically similar to other anxiety disorders, especially social anxiety disorder (Driessen et al., 2020; Gensthaler et al., 2016b), difficulties in emotional regulation (Melfsen et al., 2022), and deficits in social skills (Cunningham et al., 2004). Driessen et al. (2020) also found selective mutism to be comorbid with specific phobias and separation anxiety disorder in their meta-analytic review. Further, Sharkey and McNicholas (2012) noted that all children in their study had moderate to severe impairments in functioning, with 57% having emotional difficulties, 36% having problems with peer interaction, and 14% having behavior difficulties. As a result, children with selective mutism can perform poorly in an academic sense and can have a difficult time making friends, thereby negatively impacting basic speech, language, and social communication development starting at an early age (Cunningham et al., 2004).

The diagnosis of selective mutism is typically given once a child enters school and is often noted as a result of the child’s obvious failure to speak when speaking is expected (APA, 2022). Certainly, the consequences of selective mutism are quite noticeable once children start school, as this setting involves so many speech- and language-related activities. Nonetheless, symptoms involving failure to speak are often present prior to 5 years of age and may be recognizable as early as 2 years of age (APA, 2022; Viana et al., 2009). This lower bound at 2 years of age coincides with the age at which most children are speaking two-to-four-word sentences (Luinge et al., 2006). Much less is known about selective mutism in very young children, however, as it is difficult to gauge symptom presentations in this age group, especially when they are not attending school or in settings with other very young children. Research regarding early identification of selective mutism is sparse and dated, with a few case studies detailing young children who were 2 years of age and failing to speak to anyone aside from their immediate family (Wright et al., 1985). Behavioral symptoms linked to selective mutism are often overlooked in early childhood as typical toddler behavior (Stormont et al., 2015). Nonetheless, early childhood researchers and infant mental health specialists have argued the importance of describing and classifying disorders in very young children who are 5 years of age and younger (Osofsky et al., 2017; Osofsky & Lieberman, 2011; Zero to Three, 2016). Thus, there is a need for further understanding of selective mutism in very young children.

### 2.1. Diagnostic Adaptations for Very Young Children: The DC: 0-5

In an effort to increase awareness of emotional and behavioral symptoms in very young children, Zero to Three (1994) developed the *Diagnostic Classification of Mental Health and Developmental Disorders of Infancy and Early Childhood* (*DC: 0–3*). The *DC: 0-3* was a novel tool used to describe specific characteristics of early problems and to diagnose mental and developmental disorders in infants and toddlers. The *DC: 0-3* was later revised (*DC: 0-3R*; Zero to Three, 2005) to extend the depth and criteria included in the original *DC:0-3* in conjunction with the research and clinical practice that had occurred since its initial publication. Even with the availability of the *DC*, clinicians were still encouraged to refer to the *DSM* or the *ICD* when diagnosing very young children (Risholm Mothander, 2016), despite the developmental constraints of these systems. It is important to recognize that symptoms can present differently in infancy and toddlerhood relative to those in other age groups.

Zero to Three’s (2016) most recent *Classification of Mental Health and Developmental Disorders of Infancy and Early Childhood (DC: 0–5)* includes diagnostic criteria for selective mutism in very young children. In addition to the *DSM-5-TR*’s prominent symptom of a child’s failure to speak in settings where there is an expectation for speech (i.e., despite speaking in other situations), the *DC: 0-5* adds the specific example of preschool as a social setting where there is an expectation for speech. The *DC: 0-5* also acknowledges that a failure to speak in very young children is not accounted for by an unfamiliarity or inability to speak the language being spoken. The *DC: 0-5* specifies that the symptoms must have a significant impact on the child and family’s functioning, as evidenced by distress in the child as well as through interference with the child’s relationships. The disturbance also limits the child’s participation in developmentally appropriate activities, the child’s ability to learn and develop skills, and the family’s everyday activities or routines (Zero to Three, 2016). There have been few changes in these diagnostic criteria across the different *DC* editions; however, the age range for *DC* diagnoses has been extended from 0 to 3 years to 0 to 5 years.

Although the diagnostic features in the *DC: 0-5* are similar to those mentioned in the *DSM-5-TR*, they offer greater insight into how symptoms of selective mutism may present in very young children. Specifically, according to the *DC: 0-5* (Zero to Three, 2016)*,* selective mutism is a distressing and often severe anxiety disorder with symptoms that may not be recognized as anxiety in very young children. For instance, associated features of selective mutism in very young children may include flat facial expression, extreme shyness, tantrums, difficulty with change or sleep, and irritable mood as signs of anxiety (Zero to Three, 2016). Given that these behaviors may be overlooked as typically occurring in very young children, monitoring the severity and pervasiveness of such behaviors may allow for them to be recognized as potential signs of anxiety and target symptoms for early intervention. As a result, the *DC: 0-5* is an essential tool for professionals working with infants, young children, and their families, as it guides evaluation and treatment planning for our youngest children in ways that other classification systems do not.

Beyond the *DC: 0-5*, early childhood researchers and infant mental health specialists have the benefit of knowledge in early childhood constructs. One such construct of interest to selective mutism is temperament. Temperament can be conceptualized as a child’s behavioral style or emotional disposition that is innate, relatively consistent over time, and present across different contexts (Bates, 2001). Temperament is generally associated with individual differences in a child’s emotional, motor, and attentional reactivity as well as their self-regulation (Rothbart & Bates, 1998). Temperament characteristics, such as behavioral inhibition (when facing novel situations in general), shyness (inhibition during social situations), and fear, are widely noted to be risk factors for later anxiety disorders (Chronis-Tuscano et al., 2009; Hirshfeld-Becker et al., 2007; Volbrecht & Goldsmith, 2010). In particular, behavioral inhibition has received specific support as a precursor for selective mutism, with a connection between behavioral inhibition and lifetime diagnoses of selective mutism being documented (Gensthaler et al., 2016a). Behavioral inhibition has also been shown to predict symptoms of selective mutism, with 3-to-6-year olds with more inhibited temperament using fewer words during speech tasks (Muris et al., 2016). Screening broadly for these temperament characteristics can help capture an understanding of risk for later anxiety disorders (Abel et al., 2024). Thus, developmental knowledge adds greatly to that gleaned from diagnostic classification systems.

### 2.2. Differentiating Selective Mutism from Language Issues

In early editions of the *DSM* series (i.e., prior to *DSM-5*), selective mutism was categorized under ‘Other Disorders of Infancy, Childhood, and Adolescence.’ Research highlighting the connection between selective mutism and anxiety led to its reclassification as an anxiety disorder in the *DSM-5* (APA, 2013; Viana et al., 2009). The *DSM-5-TR* (APA, 2022) diagnostic criteria specify that the inability to speak cannot be attributed to a lack of knowledge or discomfort with the spoken language required in the particular social context in question or better explained by a communication disorder (e.g., language disorder, childhood-onset fluency disorder). With extrapolation to very young children, it should be assumed that the inability to speak cannot be attributable to the lack of language capacity and/or speech skill acquisition that would be expected at a specific developmental age. As a result, early childhood researchers and infant mental health specialists should become very familiar with the typical developmental timing of language milestones. The language capacity and speech skill acquisition of very young children should be considered closely as part of the differential diagnosis.

When it comes to distinguishing between selective mutism and communication disorders, however, the key lies in the pattern of impairment. In other words, children with only selective mutism demonstrate age-appropriate language abilities but experience anxiety-driven inhibition with their speech in specific contexts, whereas children with only a communication disorder exhibit persistent speech difficulties with verbal communication across all contexts (e.g., APA, 2022). Despite this diagnostic distinction, research indicated that mild language difficulties or delays frequently co-occur with selective mutism. Multiple studies have documented elevated rates of speech and language problems in children with selective mutism. For example, Elizur and Perednik (2003) found that speech problems were five times more prevalent in children with selective mutism relative to controls, thereby affecting approximately half of their clinical sample. This finding aligns with earlier work by Kolvin and Fundudis (1981), who also reported delayed speech onset and speech difficulties in half of their selective mutism sample. Similarly, Kristensen (2000) found that half of the children with selective mutism met *DSM-IV* criteria for one or more communication disorders compared to approximately one-tenth of controls. Other studies have reported comorbidity rates ranging from 38% to nearly 50% for speech or language disorders in children with selective mutism (Oerbeck & Kristensen, 2008; Steinhausen & Juzi, 1996).

Research has also identified specific language vulnerabilities in children with selective mutism. Studies have documented lower performance on receptive language measures, including auditory–verbal memory span (Kristensen & Oerbeck, 2006) and receptive vocabulary measures (Nowakowski et al., 2009). Studies directly comparing children with selective mutism versus social phobia have revealed important distinctions in language functioning. Although children with these conditions scored similarly on measures of anxiety, those with selective mutism consistently demonstrated greater language difficulties (Manassis et al., 2003; Yeganeh et al., 2003). This pattern of greater language impairment in selective mutism compared to other anxiety disorders has been replicated in subsequent research (Manassis et al., 2007).

These findings underscored the importance of careful diagnostic consideration of communication disorders in children who meet criteria for selective mutism. Although selective mutism’s reclassification as an anxiety disorder reflects its primary etiology in social anxiety, the high prevalence of concurrent language difficulties noted above necessitates careful consideration in clinical practice. The differential diagnosis includes several communication disorders, including language disorder (i.e., broad impairments in language acquisition and usage), speech sound disorder (i.e., articulation difficulties), childhood-onset fluency disorder (i.e., disrupted speech patterns), and social (pragmatic) communication disorder (i.e., deficits in social communication; APA, 2022). Unlike selective mutism, however, these conditions are present consistently across social contexts. This nuanced understanding of the overlap between selective mutism and communication disorders has implications for both assessment and intervention approaches, emphasizing the importance of comprehensive speech and language evaluation in clinical practice, even within a framework of anxiety-focused treatment.

### 2.3. Evidence-Based Interventions for Selective Mutism

Despite its categorization as an anxiety disorder, typical intervention models for anxiety disorders may not be appropriate for selective mutism due to its early age of onset and related symptomology. For example, with regard to psychological interventions, traditional cognitive–behavioral therapy (CBT) may not be suitable due to limitations in very young children’s cognitive development (Bergman, 2013). Further, although pharmacological interventions (e.g., SSRIs) have some supporting evidence, these are often not preferred by parents of very young children, as parents often have concerns regarding potential side-effects and medicinal dependence, especially in very young children (Hipolito et al., 2023; Manassis et al., 2016; Østergaard, 2018). Consequentially, other psychological interventions have been favored for selective mutism.

Evidence-based psychological intervention models for selective mutism are commonly categorized into three general categories: psychodynamic, systems, and behavioral approaches (Cohan et al., 2006; Steains et al., 2021). Psychodynamic approaches view selective mutism as a result of the child’s inner conflict and promote play therapy interventions (Esposito et al., 2017; Steains et al., 2021). Next, the systems approach focuses on the familial relationships that maintain the child’s symptoms and highlights the importance of psychoeducation, skills training, and collaboration (Steains et al., 2021). Finally, behavioral approaches for selective mutism are based on classical and operant conditioning as well as observational learning (Stone et al., 2002). As such, selective mutism is conceptualized as a learned behavior by which the child uses their selective mutism as a strategy to reduce the anxiety that is induced by their environment. In all, modern interventions for selective mutism have become increasingly multidisciplinary and integrate psychodynamic, systems, behavioral, and cognitive principles. As meta-analyses have consistently found that behavioral interventions tend to have larger effect sizes and outperform nonbehavioral interventions in general (e.g., Weisz et al., 1987, 1995) and, specifically, for anxiety disorders (Zhou et al., 2019), interventions for selective mutism with cognitive–behavioral components will be examined here.

## 3. Individual Interventions

Among interventions for selective mutism, integrated behavioral interventions have been particularly recognized, as such interventions utilize techniques such as contingency management, cognitive restructuring, graduated exposure, psychoeducation, and positive reinforcement (Steains et al., 2021). Collaboration between parents, teachers, and healthcare providers is emphasized, with such collaboration being especially important for very young children. Such models have received empirical support, as children (who were 4 to 8 years of age) who participated in integrative behavioral psychological interventions showed significant increases in speaking behaviors (with 75% of treatment responders rated as improved by blind independent evaluators; Bergman et al., 2013). Further, children (who were 3 to 9 years of age) showed significant increases in speech after three months of treatment, with younger children showing greater improvement (Oerbeck et al., 2014).

Specifically, Bergman (2013) developed an integrative behavioral intervention to treat children with selective mutism. This approach identifies diagnostic procedures, assessment methods, and treatment session details. Although manualized interventions are common for other anxiety-related disorders, Bergman’s (2013) integrative behavioral approach to selective mutism is relatively unique. The first two sessions are focused on rapport building, psychoeducation, and exploration of the child’s school-based interactions. Following these sessions, graduated exposure is introduced and continues throughout the remaining sessions. The last five sessions focus on the transfer of control training with the child and parents as well as relapse prevention. This approach has received empirical support and was found to significantly improve clinical symptoms of selective mutism by the last session (Bergman, 2013). Additional research is needed to better understand the efficacy of this intervention among broader populations of children, particularly very young children.

Although standard CBT may not be appropriate to treat selective mutism when there is an early age of onset, adapted CBT interventions, such as modular CBT (MCBT), have received some empirical support (Christon et al., 2012; Lang et al., 2016). MCBT was originally designed as an intervention for childhood anxiety disorders. Nonetheless, it allows for increased flexibility to meet the needs of the child and to address the unique issues of selective mutism, such as its comorbidity with other anxiety disorders (Reuther et al., 2011). MCBT uses several modules to guide treatment, including psychoeducation, physiological training, cognitive training, behavioral training, parenting training, and educational training (Chorpita, 2007). MCBT has been found to be an efficacious treatment for children who are 7 to 13 years of age with anxiety disorders (Chorpita et al., 2004). With regard to selective mutism, a study conducted by Lang et al. (2016) found that MCBT improved symptomology, with an 84% recovery rate. In addition, a case study of a 15-year-old girl with selective mutism described how MCBT was effective in increasing frequent speaking behavior (Christon et al., 2012). Further, a case study of an 8-year-old boy with selective mutism found that MCBT was effective at reducing internalizing and externalizing behavior problems, resulting in the boy no longer meeting the *DSM-IV-TR* criteria for selective mutism or social phobia (Reuther et al., 2011).

Although limited, these investigations provide promising findings for MCBT as a treatment approach for selective mutism. Due to the lack of inclusion of very young children, additional research is needed. To address the needs of very young children who are 5 years of age and younger, developmentally appropriate adaptations to MCBT would be needed to account for potential speech and cognitive levels in this age group. Specifically, increased parental support is paramount in CBT-based treatments for very young children with anxiety disorders (Hirshfeld-Becker et al., 2008). As such, MCBT treatment for very young children with selective mutism must be paired with strong parental involvement. Specifically, parents should be actively involved in the treatment process through engagement in behavioral techniques, such as modeling and shaping, when their very young child is in novel environments (Hirshfeld-Becker et al., 2008). With such adaptations, parents could learn how to appropriately manage the symptoms exhibited by their very young child as well as collaborate with their clinician to ensure that treatment generalizes beyond the therapeutic environment.

Because many treatments for selective mutism have extended intervention periods, social communication anxiety treatment (S-CAT) was developed by Shipon-Blum (2015) to provide a brief, multimodal approach. S-CAT is an integrated CBT approach and designed to increase children’s social confidence, social engagement, and verbal communication. Common strategies used in S-CAT include systematic desensitization, modeling, and positive reinforcement. In addition, S-CAT takes a holistic approach by exploring the etiology and maintenance of the child’s symptoms of selective mutism while helping the child develop appropriate coping skills. There are various forms of S-CAT, such as individualized intensive treatment (which can be conducted in a one-day or multi-day program with additional consultations in the following weeks and months). For older children, there are in-person and virtual group treatment options.

S-CAT received preliminary support following a pilot study, as 95% of children had significant increases in speaking frequency in various settings as well as with new individuals (Klein et al., 2017). The children in this study ranged in age from 5 to 12 years, making it unclear as to whether S-CAT would be an effective treatment for very young children. Nonetheless, S-CAT is marketed as a treatment for children as young as 3 years of age (Shipon-Blum, 2015). To better understand the efficacy of S-CAT for very young children, additional studies are needed. Like other CBT-based models, potential adaptations for very young children may be needed, such as increased parental support throughout treatment, parent training, and an extended intervention period.

Beyond MCBT and S-CAT, an adapted version of Parent–Child Interaction Therapy was developed for use with young children with selective mutism (PCIT-SM; Carpenter et al., 2014). The goal of PCIT-SM is to increase verbal behaviors in young children, decrease their avoidant strategies, and limit maladaptive parental behaviors (e.g., accommodation of their young child’s lack of verbalization) using CBT components. Somewhat different from PCIT proper, there are two primary phases in PCIT-SM: child-directed interaction (CDI) and verbal-directed interaction (VDI). The CDI phase resembles traditional PCIT but has a greater emphasis on the young child’s speaking behaviors. During this phase, the parent–child relationship is strengthened through the rewarding of positive behaviors (e.g., providing praise for speech; Carpenter et al., 2014). In addition, parents are taught to selectively attend to their young child’s behaviors and to limit ‘mind reading’ (i.e., anticipating their young child’s desires before verbalization; Kurtz, 2015) so that the young child has to speak to gain their desire.

Once the young child can respond to probing questions during the CDI phase, the VDI phase is introduced. VDI is akin to parent-directed interaction (PDI) in PCIT proper but has a focus on generalization of the young child’s speech with new people in novel environments (Cotter et al., 2018). This generalization is accomplished through question-based practice, with parents using forced-choice and open-ended questions paired with contingent secondary reinforcement and graduated exposure to different settings, individuals, and activities (Carpenter et al., 2014). Although PCIT-SM has not received randomized empirical testing, findings suggest that it fosters increases in spontaneous speech and verbal responses in young children who range in age from 4 to 10 years (Catchpole et al., 2019; Mele & Kurtz, 2013). PCIT-SM treatment also demonstrated the maintenance of treatment outcomes at 3-month and 1-year follow-ups (Catchpole et al., 2019). Additional randomized empirical investigations are needed, especially with very young children who are 5 years of age and younger.

## 4. Group Interventions

Group-based integrative approaches have also gained recognition, as they provide an opportunity to practice exposure in a peer setting. Notably, intensive group behavioral treatment (IGBT) has been increasingly utilized among clinicians and was developed based on the foundations of PCIT and CBT. IGBT occurs across one to two weeks during the summer to prepare the child for the upcoming school year and focuses on graduated exposure and structured reinforcement (Lorenzo et al., 2021). A key component of IGBT includes all-day group sessions referred to as ‘camp.’ During these group sessions, groups of six to twelve children with selective mutism are placed in a classroom-like setting. Each child has an individual staff counselor to assess each child’s progress and deliver more formal intervention skills, such as child-directed interaction (Lorenzo et al., 2021). Due to its duration, this form of intervention may be more accessible and preferred by families.

Although additional research is needed, current investigations on IGBT have reported that it effectively reduces symptoms of selective mutism (Cornacchio et al., 2019; Hong et al., 2023; Kupferberg et al., 2024). Nonetheless, studies examining mostly older children (5 to 9 years of age and 3 to 10 years of age, respectively) with selective mutism noted that IGBT may need adaptations for it to be suitable for very young children. These adaptations may include the implementation of an individualized reward system, simplified psychoeducation, daily debriefing with children and parents, increased social skills training, and adapted interpersonal staff communication (Cornacchio et al., 2019; Lorenzo et al., 2021). Very young children would also require more oversight and support in the classroom-like camp setting as well as more parent–staff interfacing.

## 5. Overall Findings Summarized

Research investigating the efficacy of nonpharmacological interventions for selective mutism may be limited to only two known meta-analyses (based on the authors’ knowledge; Hipolito et al., 2023; Stone et al., 2002). These studies revealed consistent findings for the efficacy of integrative behavioral approaches for the treatment of selective mutism. Moreover, both meta-analyses concluded that, in comparison to no treatment, psychological intervention was more effective at reducing the symptoms of selective mutism, with significant and large effect sizes. Since 1992, however, there have only been six randomized controlled trials assessing the efficacy of psychological interventions for the treatment of selective mutism. There are also no standardized outcome measures for selective mutism, making it difficult to draw conclusions from comparative investigations, such as meta-analyses (Creswell et al., 2021). As such, further conclusions regarding specific interventions cannot be made (Hipolito et al., 2023). Thus, there is a crucial need to establish standardized outcome measures for selective mutism and expand sound experimental studies in this area, especially for very young children.

### Intervention Adaptations to Consider for Very Young Children

As already suggested above, traditional interventions for selective mutism may be adaptable for use with very young children. Generally, when considering specific modifications or accommodations for very young children with selective mutism, it is particularly important to be aware of each child’s unique needs and in what setting interventions will be provided. Of particular note, intervention strategies must be adapted to fit the social and cognitive capabilities of very young children.

## 6. Home-Based Adaptations

At home, parents and caregivers play an imperative role in reducing anxiety and fostering communication. Since very young children may not yet have developed strong verbal skills, parents should encourage both verbal and nonverbal methods of communication (e.g., gesturing, pointing; Hung et al., 2012). In particular, parents can support early language development through parallel talk (i.e., the act of narrating daily activities). For example, when getting ready for school, parents should simply tell their child what they are doing. By modeling vocalizations, parents are introducing their child to speech as well as demonstrating how natural communication looks. Parallel talk and the modeling of vocalizations are especially helpful for children with selective mutism, as these strategies do not demand or expect verbal responses (Dow et al., 1995). Play-based approaches can also promote early speech and language development as well as comfort with speech and language production. Interactive games (e.g., peekaboo, nursery rhymes, animal sounds) provide opportunities for verbal communication in low-stress, low-pressure environments (Kovac & Furr, 2018). Finally, parents should also be encouraged to incorporate baby sign language as an alternate means of self-expression while speech develops (Dow et al., 1995; Mohajerin et al., 2023).

After very young children have been gradually exposed to verbal communication, they should then be exposed to new social environments. Age-appropriate activities (e.g., playdates, parks with other children present, childcare) should be introduced. Since selective mutism is characterized by anxiety, children should be permitted to bring along transitional or comfort objects (e.g., a favorite toy or blanket) to provide additional security and comfort (Dow et al., 1995). By removing stress and adding comfort, very young children are afforded chances to communicate in ways that feel natural to them.

When working with therapists, it is recommended that the therapist and very young child interact in spaces where the very young child is most comfortable (e.g., at home). Such locations minimize any pressure associated with speaking. Other adaptations and strategies include integrating the very young child’s strengths, likes, and dislikes into their intervention program, being sensitive to what activities interest them, and encouraging them to talk about school or other outside settings while at home. Research also suggested implementing various environmental interventions to make very young children more comfortable with their surroundings (e.g., have a trusted individual accompany the child to school and participate in classroom/school activities, create quiet corners, or employ visual aids; Hung et al., 2012).

## 7. Classroom-Based Adaptations

When working with very young children with selective mutism in classroom settings, teachers are advised against pressuring them to speak, as such pressure will only increase anxiety (Marc & Crundwell, 2006). Instead, classroom-based interventions require a multidisciplinary approach. Cooperation among parents, teachers, administrators, and other support staff contributes to more effective intervention efforts. This team must work together to implement intervention efforts that not only increase very young children’s speech in school but address their distress associated with speaking (Hudson et al., 2023; Marc & Crundwell, 2006).

Classroom strategies for teachers of very young children with selective mutism are most effective when combined with professional guidance and parental assistance. Kovac and Furr (2018) suggested several techniques to improve student comfort. These techniques include contingency management (i.e., a style of positive reinforcement in which the teacher encourages minor nonverbal forms of communication like nodding or pointing so that the student becomes more verbally interactive in the classroom over time) and fading (i.e., a technique where the number of individuals present or the distance between the child and other individuals is gradually increased over time while the child is speaking; Kovac & Furr, 2018).

In addition to positive reinforcement, Kovac and Furr (2018) endorsed exposure-based interventions, like exposing the very young child to increasingly anxiety-provoking situations and encouraging them to speak as they become desensitized. Finally, Kovac and Furr (2018) proposed a technique of self-modeling for classroom settings. In this technique, parents show teachers videos of the child speaking in contexts where the child is comfortable so that the teacher can see the child’s speech and language capabilities and so the child can view the videos of themselves speaking while they are in the classroom setting (Kovac & Furr, 2018).

For very young children, gradual social exposure is key to lessening anxiety and increasing comfort. Teachers and caregivers should allow very young children to observe group interactions *before* they are expected to engage directly, thereby encouraging familiarity with their new classroom environment. Parallel play (i.e., where children play alongside their peers rather than engaging directly) can ease social anxiety and create opportunities for very young children to speak naturally. In addition, nonverbal participation should be constantly reinforced. Actions such as gesturing, pointing, and clapping should be permitted as valid social responses (Dow et al., 1995; Mohajerin et al., 2023).

Early childhood educators and childcare providers should allow parents and caregivers to be present during initial transitions into childcare/school settings (Hudson et al., 2023). They also should make efforts to incorporate music, clapping games, and movement-based activities to encourage natural speech. During storytelling, educators should use puppets and exaggerated facial expressions to keep nonverbal children engaged in classroom activities. Activities such as blowing bubbles and imitation games support early oral motor development, which is foundational for proper speech and language development. Vocal group activities (e.g., singing or call-and-response games) are also helpful. Through this array of activities, speech is not directed at an individual person but rather the entire class (Hudson et al., 2023; Mohajerin et al., 2023). Overall, no matter the setting, parents, teachers, and others are strongly encouraged to exercise patience and compassion with very young children with selective mutism. These different adaptations should focus on reducing pressure, fostering gradual social exposure, and reinforcing both verbal and nonverbal communication.

## 8. Conclusions

Understanding and addressing selective mutism in young children requires adaptations in both the diagnostic and intervention stages. As symptoms are most commonly recognized when very young children enter school, selective mutism often goes unnoticed in very young children who are younger than 5 years of age, especially in the context of current *DSM-5-TR* classification and conceptualization. To better assess selective mutism in very young children, the *DC: 0-5* criteria provide clarity by highlighting the presentation of symptoms in this age group, including associated features related to facial expressions, behavior, and mood. Thus, utilization of the *DC: 0-5* is encouraged for a more comprehensive assessment of and intervention for selective mutism in very young children. Further, it is important to distinguish selective mutism from language disorders in children so that appropriate intervention can be provided. Primarily, very young children with selective mutism have developmentally appropriate language abilities but intentionally inhibit themselves in anxiety-inducing situations. This differential symptomatology is distinct from language disorders, as the very young child’s functional ability to communicate is not inherently impaired, although language delays may co-occur (Oerbeck & Kristensen, 2008). To treat selective mutism, evidence-based models have been developed from an integrated behavioral approach and have been shown to significantly reduce clinical symptoms (Bergman, 2013; Lorenzo et al., 2021). As evaluative research is limited due to the lack of randomized controlled trials and standardized outcome measures (Hipolito et al., 2023), more research is needed, especially with very young children. Lastly, adaptations to interventions for very young children that focus on their environment, developmental abilities, and relationships are crucial.

## Data Availability

Not applicable.
